# Fluid Shear Stress Regulates the Invasive Potential of Glioma Cells via Modulation of Migratory Activity and Matrix Metalloproteinase Expression

**DOI:** 10.1371/journal.pone.0020348

**Published:** 2011-05-26

**Authors:** Henry Qazi, Zhong-Dong Shi, John M. Tarbell

**Affiliations:** Department of Biomedical Engineering, City College of New York, City University of New York, New York, New York, United States of America; Virginia Commonwealth University, United States of America

## Abstract

**Background:**

Glioma cells are exposed to elevated interstitial fluid flow during the onset of angiogenesis, at the tumor periphery while invading normal parenchyma, within white matter tracts, and during vascular normalization therapy. Glioma cell lines that have been exposed to fluid flow forces *in vivo* have much lower invasive potentials than *in vitro* cell motility assays without flow would indicate.

**Methodology/Principal Findings:**

A 3D Modified Boyden chamber (Darcy flow through collagen/cell suspension) model was designed to mimic the fluid dynamic microenvironment to study the effects of fluid shear stress on the migratory activity of glioma cells. Novel methods for gel compaction and isolation of chemotactic migration from flow stimulation were utilized for three glioma cell lines: U87, CNS-1, and U251. All physiologic levels of fluid shear stress suppressed the migratory activity of U87 and CNS-1 cell lines. U251 motility remained unaltered within the 3D interstitial flow model. Matrix Metalloproteinase (MMP) inhibition experiments and assays demonstrated that the glioma cells depended on MMP activity to invade, and suppression in motility correlated with downregulation of MMP-1 and MMP-2 levels. This was confirmed by RT-PCR and with the aid of MMP-1 and MMP-2 shRNA constructs.

**Conclusions/Significance:**

Fluid shear stress in the tumor microenvironment may explain reduced glioma invasion through modulation of cell motility and MMP levels. The flow-induced migration trends were consistent with reported invasive potentials of implanted gliomas. The models developed for this study imply that flow-modulated motility involves mechanotransduction of fluid shear stress affecting MMP activation and expression. These models should be useful for the continued study of interstitial flow effects on processes that affect tumor progression.

## Introduction

Developing glioma vasculature is convoluted with temporally and spatially heterogeneous flow and enhanced neovascularization [1]–[5]. Angiogenesis-induced breakdown of normal vasculature leads to hyperpermeable vessels that are associated with elevated interstitial convection into the parenchyma and consequently elevated fluid shear stress on tumor cell surfaces [6]–[13]. Solid brain tumors are also characterized by elevated fluid flux into the parenchyma at the tumor boundary [13]. Interstitial fluid in the brain eventually drains through white matter tracts into cerebrospinal fluid or into the subarachnoid space [6], [10]. It should be noted that since the central nervous system does not have ‘true’ lymphatic vessels, enlarged tumors in the brain lead to edema and flow velocities come to a near halt unless antiangiogenic therapy is applied [6].

Normalization of the tumor vasculature via antiangiogenic interventions decreases the fluid flow heterogeneity to improve fluid drainage through the parenchyma and white matter tracts [1], [2], [13], [14]. Moreover normalization of tumor vasculature alters the intratumor interstitial flow rates thereby modifying shearing forces on cells throughout the tumor [13]. In spite of the aforementioned characteristics, the contributions of the fluid dynamic microenvironment and the effect of normalization on the migratory activity of tumor cells have been largely overlooked. There have been no assessments of the effect of fluid shear stress on the migratory activity of glioma cells. It has, however, been theorized that spatial and temporal heterogeneities in flow, elevated fluid flow at the periphery, and fluid shear stress may modulate metastasis, growth, and invasion [1], [15], [16].

The defining step of cell invasion into normal tissue is the degradation of the extracellular matrix (ECM), within and around the tumor, by the activity of matrix metalloproteinases (MMPs) [17]–[21]. The enhanced expression of proteases by gliomas indicates that MMPs play a major role in tissue invasion and degradation of the extracellular matrix [22]. Many MMP genes are susceptible to modulation by extracellular stimuli and fluid shear stress might be one such stimulus [23]. Since MMP expression and activity are modulated by fluid shear stress in various (non-tumor) cell types [24]–[26], shearing forces could regulate the migratory behavior of glioma cells. Therefore any observed modulations of MMP expression in this study may be reflective of migratory activities and invasive potentials.

Modified Boyden chamber models have proven to be an effective way to analyze the migration response of glioma cells to a variety of stimuli [20], [23], [27]. One study utilized a modified Boyden chamber to demonstrate that flow-induced chemokine gradients lead to directional migration of cells [28]. The present study attempts to show that in addition to the previously recognized extrinsic roles of fluid flow, shear stress can modulate intrinsic characteristics of cells thus altering their motility and invasive potential. This study utilizes a three-dimensional modified Boyden chamber to model the effects of fluid shear stress on the motility of tumor cells.

Another motivation for this study was to identify shear stress as a key regulator of motility that may explain discrepancies between *in vitro* and *in vivo* invasiveness of glioma cell line models. Several *in vitro* studies claimed that U87 cells exhibited one of the highest migratory activities among glioma cell line models and displayed biological properties and characteristics similar to human glioblastomas obtained through surgical interventions [4], [20], [21]. Contrary to these findings, *in vivo* studies have shown that the U87 cell line is minimally to non-invasive and lacks pseudopalisading unlike the CNS-1 and U251 cell lines [29]. Therefore we investigated the influence of shear stress on the motility of these cells and demonstrated that the invasive potentials of these three cell lines were altered differently by shearing forces.

## Materials and Methods

### Cell culture and chemoattractant

U87 human glioma (HTB-14; ATCC), rat CNS-1 glioma (Dr. William F. Hickey and Dr. David J. Graber, Dartmouth Medical School), and U251 human glioma (Dr. Eric C. Holland, Memorial Sloan-Kettering Cancer Center) cell lines were cultured in DMEM (Sigma) supplemented with 10% FBS (HyClone) and 1% Penicillin/Streptomycin (Sigma). Cells were grown to a minimum of 70% confluence, and then all experiments were conducted in humidified incubators. TGF-α (Sigma) was chosen as the chemoattractant since it is one of the most potent stimulators of migration [27]. The optimal concentration of TGF-α was determined via Boyden chamber experiments (data not shown); ultimately, 10 nM TGF-α in DMEM without serum was added to the companion wells for all invasion assays unless otherwise specified.

### Three-dimensional (3D) cell suspensions

**Figure 1:**
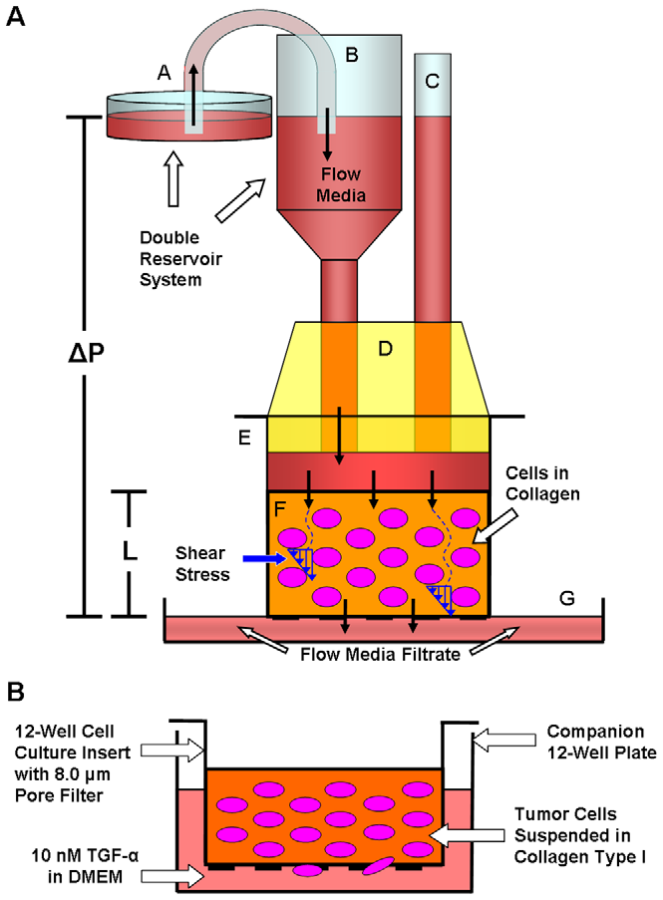
Darcy Flow Experimental Apparatus for the application of shear stress in 3D and Modified Boyden chamber invasion assay. (**A**) The flow apparatus applied a constant hydrostatic pressure via a double reservoir system composed of a larger reservoir [A] feeding flow media into the smaller syringe reservoir [B] and a pressure release tube [C], both of which were fastened within a rubber seal [D] to the cell culture insert [E]. The pressure release tube [C] ensured that there were no fluctuations in pressure applied across the collagen/cell suspension [F]. Hydrostatic pressure [ΔP] drove media throughout the thickness of the gel [L] and exerted shear stress on the cell membranes. Flow media filtrate was collected in another reservoir [G]. (**B**) At the end of the flow period, the inserts containing the cell suspensions [E] were decoupled from the apparatus and the invasion assay ensued. During the migration period, 10 nM TGF-α directed cells to migrate through 8 µm pores towards the underside of the filter. At the end of the migration period, cells on the underside of the inserts were stained and migration rates were quantified.

The boundaries between brain tumors and parenchyma have been shown to contain interstitial collagen type I among other matrix components, and some implanted glioma cell lines form interstitial ECM that consists primarily of interstitial collagen [20], [23], [30]. A model was developed in which glioma cells suspended in type I collagen were exposed to 3D fluid shear stress via the Darcy Flow Experimental Apparatus (Fig. 1*A*). High concentration Rat Tail Collagen Type I (BD Biosciences) was utilized as the stock and all dilutions were carried out using serum-containing media (DMEM containing 10% FBS; culture media). The walls of 12-well cell culture inserts (containing 8.0 µm pore filters [BD Falcon]) were pre-coated with 50 µl of 1 mg/ml collagen to prevent gel detachment or contraction by the suspended cells. 50,000 cells were suspended in 400 µl of 2 mg/ml collagen and immediately incubated within the pre-coated culture inserts for proper gelation to reduce settling of cells at the bottom due to gravity. The cell/collagen suspensions were incubated for 12 hours with 800 µl of serum-containing media in each well to allow sufficient time for cell spreading.

### Three-dimensional (3D) shear stress model

The three-dimensional model simulated the interstitial flow forces that cells would encounter within the interstitium in order to study the effects of shear stress on chemotactic migration (Fig. 1*A*). DMEM with 10% serum was used as the flow media, and both time of exposure to flow (up to four hours) and shear stress levels were varied. The shear stress (*τ*) on the cell surface was calculated knowing the Darcy permeability coefficient (*K_p_*) of the 3D collagen/cell suspension and assuming cylindrical or spherical cell geometries based on theory by Wang and Tarbell [31], and Brinkman [32]: 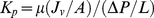
(1)

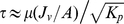
(2)where *µ* is the fluid viscosity, *J_v_* is the volumetric flow rate, *A* is the area of the filter, *L* is the thickness of the gel, and Δ*P* is the pressure drop [17], [31], [32]. The shear stress equation is an approximation; for spherical cells it should be multiplied by π/3, and for cylindrical cells by a factor of π/4 [31]. These model equations were also utilized to determine the physiologic range of shear stress based on established permeability and interstitial flow velocity measurements in developing tumors and in brain tumors.

During the flow period, the fluid filtrate was quantified every 10 minutes after initiation of flow through the gel to determine the volumetric flow rate, *J_v_*. After it was determined that *J_v_* was stable after the first 10 minutes of flow, when the collagen compacts to a fixed point (discussed below), then the filtrate was measured on an hourly basis. The flow properties of the collagen/cell suspension along with the parameters characterizing the 3D model are presented in Table 1.

**Table 1:** Flow Properties of Collagen Gel and Cell Suspensions.*

Pressure, Δ*P* (cm H_2_0)	Flow Rate, *J_v_* (µl/min)	Velocity (µm/sec)	Permeability, *K_p_* (10^−15^ m^2^)	Shear Stress, τ (dynes/cm^2^)^#^
1.0	4.32±0.42	0.83±0.08	4.16±0.41	0.11±0.01
5.0	9.58±1.18	1.85±0.23	1.84±0.23	0.36±0.02
7.0	15.97±9.89	3.08±2.01	2.05±1.27	0.55±0.18

*Darcy permeability and shear stresses obtained by utilizing equations (1) and (2). *Viscosity (*µ*) −0.84 cP; Area (*A*) −0.865 cm^2^; Length (*L*) −600 µm.

#Each shear stress level was different from the other levels (*p*<0.005).

Data presented as mean±standard deviation.

### Compaction of cell suspensions

It became evident that the initial application of fluid flow was permanently changing the thickness of the gels by compaction of the collagen and thereby altering the cell distribution, collagen density and permeability. Therefore, following the initial 12-hour incubation period, 8 cmH_2_O of differential pressure was applied across all gels inducing flow for 10 minutes to pre-compact the gels before the flow experiment. The gels that were allowed to compact with no additional flow were designated as compacted controls. Assuming minimal collagen degradation by the cells during the initial incubation and flow periods, a mass of collagen/volume analysis was performed by collecting and measuring the volume of the gels (from both compacted controls and flow cases) to determine the collagen density of the compacted gels. During all incubation periods, to ensure that the gels would not detach from the filter, the media in the well was maintained at a level below the upper surface of the gel, a normal pressure of 0.05 cm, to prevent back flow of media and to maintain compaction of the gel under its own weight.

### Cell distribution and viability

Following the flow period, some of the 12-well inserts containing the 3D cell suspension were stained with Calcein AM (Invitrogen) and imaged using a Leica Confocal microscope. Fluorescence at different depths was obtained to determine the thickness of the gels (*L*), to quantify the effect of compaction on cell distribution (from initiation of flow), and to ensure proper attachment of the gels to the filters. Confocal imaging was repeated after a 48 hour migration period on other cell suspensions in order to observe cell morphology.

### Three-dimensional (3D) shear stress invasion assays

Following completion of the flow period, all cells on the underside of the inserts were mechanically removed to “zero” the initial migration count [26]. The inserts containing the cell suspensions were then incubated with 700 µl of 10 nM TGF-α in the well for 48 hours of migration (without flow; Fig. 1*B*). Thus the flow (shear) and migration periods were separated so that flow effects could not be interpreted as resulting from the convection of chemoattractant or signaling molecules produced by the suspended cells. At the end of the migration period, cells that had migrated to the underside of the inserts were fixed, stained with DiffQuik (Dade Behring), and counted in 5 fields to quantify migration rates; method established by Garanich et al [24]. It should be noted that cells that migrated through the pores adhered to the underside of the filter and there was no evidence of cells detaching from the filter and floating in or attaching to the bottom of the well.

### Broad-spectrum MMP inhibitor invasion assays

GM6001 (Calbiochem), a broad-spectrum MMP Inhibitor, and GM6001-NC (Calbiochem), as a negative control, were utilized to assess the extent to which the glioma cell lines within the collagen/cell suspensions depended on active MMP expression for invasion. Standard 48 hour invasion assays were conducted with the glioma cells migrating from the suspensions towards 10 nM TGF-α solutions containing 10 µM GM6001 or 10 µM GM6001-NC.

### Isolation of chemotactic motility

To confirm that autologous chemotaxis flow effects had been isolated and removed for all cell lines being used, the Darcy flow experiment and invasion assay were carried out without the use of TGF-α as a chemoattractant. Additionally, the potency of TGF-α (without flow) was determined for each cell line in the modified 3D Boyden chamber to ensure that 10 nM TGF-α was able to effectively provide a directional cue for cells to migrate so that motility effects could be established.

### Cell apoptosis and viability assay

To determine if apoptosis played a role in the invasion response brought on by flow, after the 48 hour migration period, both control gels and gels exposed to a differential pressure drop of 7 cmH_2_O were stained with the Vybrant Apoptosis Assay Kit no. 2 (Invitrogen) following the manufacturer's instructions. Gels were also stained with Calcein AM to further confirm viability after the migration period. Cell apoptosis and viability images were acquired utilizing a Nikon inverted fluorescent microscope.

### MMP activity assays

Following the migration period, the conditioned media from the wells of sheared and control (non-sheared) glioma cells were collected and stored. MMP-1 and MMP-2 are two of the most important MMPs for the degradation of the ECM in gliomas [18]. Triple helical collagen is cleaved by interstitial collagenases (mainly active MMP-1) and subsequent degradation of denatured collagen fibrils and basement membrane ECM by gelatinases (predominantly active MMP-2) [22], [33]. Changes in total (active and pro-) and active levels of MMP-1 and MMP-2 were determined utilizing the AnaSpec SensoLyte Plus 520 MMP-1 Assay Kit and the AnaSpec SensoLyte 520 MMP-2 Assay Kit (AnaSpec, San Jose, CA). Both kits used the fluorogenic substrate 5-FAM/QXL520 and upon cleavage by their respective MMP, the fluorescence intensity could be measured at 490/520-nm wavelength [26]. Manufacturer's instructions were followed to determine relative concentrations of the MMPs in the conditioned media.

### RNA extraction and isolation

Cell lysis and RNA extraction from the collagen suspension was carried out with the use of TRIzol Reagent (Invitrogen) following the manufacturer's instructions. After homogenization (decoupling of proteins from nucleic acids) insoluble matrix was removed via centrifugation as recommended by the supplier. For total mRNA isolation and purification the RNeasy Mini Kit (Qiagen) and the associated QIAvac 24 vacuum manifold (Qiagen) were utilized following the supplier's instructions.

### Reverse transcription-polymerase chain reaction

RT-PCR was performed to validate that interstitial flow was modulating MMP levels. Reverse transcription to cDNA was performed following the Cells-to-cDNA II Kit (Ambion) procedures. Quantitative real-time PCR (RT-qPCR) was performed on the ABI PRISM 7000 sequence detection system (Applied Biosystems) with the reactions containing SYBR Green PCR Master Mix (Applied Biosystems). In addition to the RT-qPCR, representative samples of the amplified mRNA products were isolated through gel electrophoresis and visualized under excitation by ultraviolet light in the presence of 0.1 µg/ml ethidium bromide in 2.75% agarose gels. It was determined that modulation of MMP-1 levels was the primary effect of shearing the U87 cells. Therefore a 274-bp MMP-1 product was amplified using the sense primer as 5′-TGA GGG GAA CCC TCG CTG GG -3′ and its antisense primer as 5′-TCC CCT CCA ATA CCT GGG CCT G-3′ (Genebank accession no. NM_002421.3), and a 267-bp GAPDH product was amplified using the sense primer as 5′-CCT GAC CTG CCG TCT AGA AA-3′ and its antisense primer as 5′- TTA CTC CTT GGA GGC CAT GT-3′ (Genebank accession no. NM_002046) [34]. It was further established that modulation of MMP-2 levels was the primary effect of shearing the CNS-1 cells. Therefore a 200-bp rat MMP-2 product was amplified using the sense primer as 5′-GAT GGA TAC CCA TTT GAC GG-3′ and its antisense primer as 5′-CTG CTG TAT TCC CGA CCA TT-3′ (Genebank accession no. NM_031054) [26], and a 232-bp GAPDH product was amplified using the sense primer as 5′-TCT TCA CCA CCA TGG AGA A-3′ and its antisense primer as 5′-ACT GTG GTC ATG AGC CCT T-3′ (Genebank accession no. NM_017008) [26]. GAPDH served as an internal control for each sample. β-actin was also used as a secondary housekeeping gene to further confirm the findings (data not shown). Specificity of the amplified products was verified by both dissociation curve analysis and by gel electrophoresis.

### RNA interference

To determine if shear-dependent modulation of MMPs could affect migration, specific MMP mRNAs were silenced by short hairpin RNA (shRNA). MMP-1 shRNA oligonucleotide corresponding to bases 305 to 323 of the MMP-1 mRNA, previously cloned into the pSuper-retro-puro expression vector (OligoEngine), was utilized to silence MMP-1 gene expression in U87 cells (shRNA designated as ‘305’-a kind gift from Dr. Constance E. Brinckerhoff, Dartmouth Medical School) [35]. As a control vector, a scrambled sequence cloned into the pSuper-retro-puro plasmid was also provided (control shRNA shMAMMX designated as ‘MMX’; another kind gift from Dr. Brinckerhoff) [35]. Both vectors were amplified utilizing XL1-Blue Competent Cells (Stratagene) and purified utilizing the QIAprep Spin Miniprep Kit (Qiagen) following the suppliers' instructions. MMP-2 shRNA Plasmid (r) and its corresponding Control shRNA Plasmid-A (Santa Cruz Biotechnology) were utilized to silence MMP-2 gene expression in CNS-1 cells. Puromycin resistance was designed into all vectors utilized in this study so that cells stably expressing the shRNA could be selected.

### shRNA transfection and puromycin selection

The shRNA plasmids were transfected into their respective cell lines using Lipofectamine LTX and PLUS reagents (Invitrogen) and cells were grown to confluence. Following transfection, cells stably expressing their respective shRNA plasmids were isolated by puromycin selection. The optimal puromycin concentration of 1.5 µg/ml was chosen such that non-transfected cells would die within 2 days of culture (data not shown). For the duration of the MMP gene knock-down experiments, tranfected cells were grown in culture media supplemented with 1.5 µg/ml puromycin.

### RNA interference invasion assay

The invasive potential of glioma cells stably expressing their respective shRNA vectors was determined. Standard invasion assays were conducted and the suspended glioma cells were allowed to migrate towards 10 nM TGF-α for 48 hours. After the migration period, cells suspended inside the gels were lysed, RNA was extracted and purified, and reverse transcription was performed as previously described. RT-qPCR was performed and representative samples for the amplified mRNA products were isolated through gel electrophoresis and visualized in the presence of ethidium bromide. Cells that had migrated to the underside of the inserts were fixed and stained with DiffQuik, and migration rates were quantified.

### Collagen and gelatin zymography

Following the migration period, the conditioned media from the wells of glioma cells transfected by the control vectors and knockdown vectors were collected and stored. Collagen and gelatin zymography were performed as detailed by Shi et al [26] to confirm knockdown of MMP expression by shRNA vectors. Collagen zymography was used to confirm knockdown of pro- and active levels of MMP-1 expression in U87 cells and gelatin zymography was used to confirm knockdown of pro- and active levels of MMP-2 expression in CNS-1 cells brought on by their respective shRNA gene silencing. MMP expressions were quantified using the Quantity One software (Bio-Rad) and were presented as percentage of their respective controls [26].

### Specific MMP inhibitor invasion assay

To further verify invasion effects by shRNA knockdown, specific MMP inhibitors were used in invasion assays with non-transfected cells. Standard 48 hour invasion assays were conducted with the glioma cells migrating from the suspensions towards 10 nM TGF-α solutions containing 10 µM of either MMP Inhibitor I or MMP-2 Inhibitor I (Calbiochem). MMP Inhibitor I was utilized at a concentration that would inhibit MMP-1 activity in U87 cells and MMP-2 Inhibitor I was used to inhibit MMP-2 activity in CNS-1 cells.

### Statistical analysis

All data were normalized by their respective controls and are presented as mean±standard error of the mean. The two-tailed Student's t-test (type 3) was utilized to determine statistical significance and Bonferroni corrections were applied for multiple comparisons (#). For cell distribution and fluorescence in the gels a two-factor analysis of variance (ANOVA) was utilized followed by a post-hoc analysis for intensity and distance.

## Results

### Effect of fluid compaction on cell distribution

**Figure 2:**
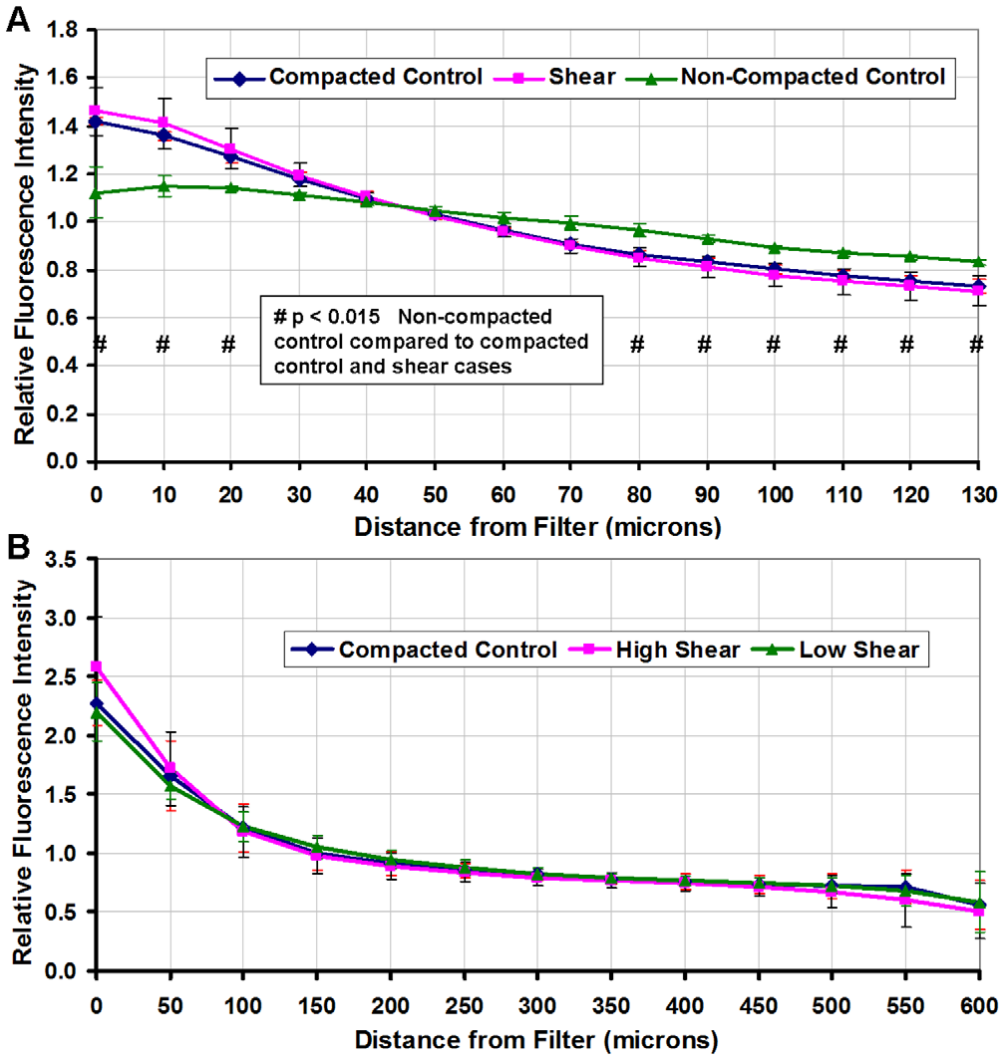
Fluorescence intensity of gels containing cells stained with Calcein to quantify cell distribution within the collagen suspension. (**A**) Fluorescence of horizontal slices (10 µm each) up to 130 microns above the filter for gels that were compacted, not compacted, and suspensions that were exposed to four hours of flow. The cell distribution in non-compacted gels was distinctive when compared to the compacted control and four-hour flow gels (# *p*<0.015). (**B**) Intensity of the 600 micron thick collagen suspensions (50 µm slices) to verify similar cell distribution of the compacted control and the experimental flow cases at 0.36 dynes/cm^2^ (low) and 0.55 dynes/cm^2^ (high) shear stress. Compacted control and flow cell suspensions utilized for the migration study had similar cell distributions (*p*≈0.8). In all cases, cells density increased towards the filter interface (*p*<0.0001). All cases were normalized to their respective average intensities and the data presented as mean±SEM.

The collagen concentration of gels exposed to flow (sheared) and compacted (non-sheared) controls was 8.09±0.26 mg/ml-4 times more concentrated than the original gels before compaction. Compaction only affected cell distribution perpendicular to the filter (vertical direction); cells remained viable, morphologically normal, and well distributed in every horizontal plane throughout the thickness of the gels (Supplementary Fig. S1). The cumulative effect of compaction by flow through the gels was most apparent closer to the filter (Supplementary Fig. S2). The cell distribution of the compacted controls and the suspensions exposed to four hours of shear were similar, whereas the distribution of non-compacted controls was different from the compacted control and sheared cases (Fig. 2*A*). Following the flow period, the cell distribution of control suspensions compacted by ten minutes of flow, were similar to gels exposed to four hours of varied shear stress (Fig. 2*B*).

### Darcy permeability and three-dimensional shear stress

The flow rates, flow velocities, Darcy permeabilities, and shear stresses for varied hydrostatic pressure drops have been reported (Table 1). For comparison, one study reported a Darcy permeability of 3.0×10^−16^ m^2^ with a collagen concentration of 45 mg/ml for glioma interstitium [17]. During the initial stages of a developing tumor, Chary and Jain reported fluid flow velocities of up to 2.00 µm/sec [36]. Other studies measured fluid flow within brain tumors and reported velocities of 0.17 to 1.39 µm/sec [9], [10], [37]. According to equation (2), the reported permeability and flow velocities suggest fluid shear stresses of up to 0.97 dynes/cm^2^ for developing tumors and a shear stress range of 0.09 to 0.68 dynes/cm^2^ for brain tumors. The experimental shear stress levels were in the range of these physiological values. It should be noted that the permeability of gels exposed to 1 cmH_2_O differential pressure drop was higher, probably as a result of recoiling of the elastic collagen gels during the flow period. However, all gels were compacted before the flow period and were the same thickness implying a similar density/permeability and sustained compaction during the migration period.

### Migratory activity suppressed by three-dimensional shear stress

**Figure 3:**
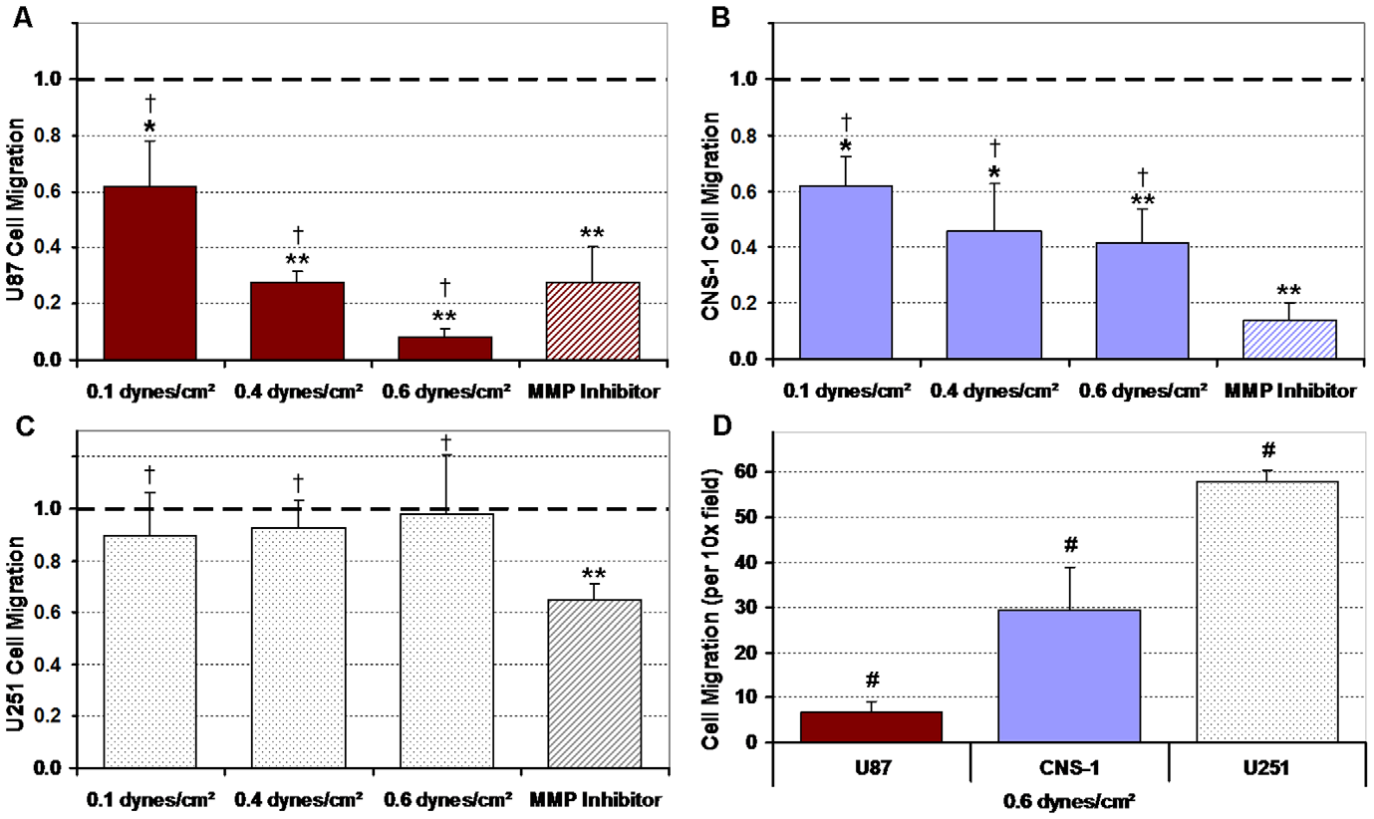
Migration response of U87, CNS-1, and U251 glioma cells after exposure to fluid shear stress. All results were normalized to non-sheared controls (1.0). The migration responses for glioma cells exposed to shear stress or the broad spectrum MMP inhibitor are presented. Exposure to four hours of 0–0.55 dynes/cm^2^ shear stress elicited a heterogeneous response in the cell lines. (**A**) In response to increasing flow the migration of the U87 cell line was suppressed the most; (**B**) migration of the CNS-1 cell line was suppressed in a similar pattern but to a lesser extent than the U87 cell line; (**C**) and the U251 cell line was the least responsive to shear. The suppression of migratory activity in response to the MMP inhibitor was not significantly different from the suppression of migration by shear stress for any cell type († *p*>0.05). (**D**) After exposure to 0.55 dynes/cm^2^ shear stress the U87 cell line was minimally invasive, the CNS-1 cell line was more invasive than the U87 cell line, and the U251 cell line was the most invasive (raw migration rates for the three cell lines are presented). Data presented as mean±SEM. Note: * *p*<0.05; # *p*<0.015; ** *p*<0.005.

In the 3D model, the migratory activity of U87 and CNS-1 cell lines was increasingly suppressed as the time of exposure to shear stress (Supplementary Fig. S3) or the level of shear stress was increased (Fig. 3) whereas U251 migration was not affected by shear. Shear stress suppressed U87 and CNS-1 migratory activity by up to 92% and 58%, respectively when compared to normalized controls (*p*<0.005). The MMP inhibitor suppressed 72%, 86%, and 35% of the U87, CNS-1, and U251 migratory activity, respectively, when compared to normalized controls (*p*<0.005), suggesting that baseline invasion was mediated by MMPs. Additionally, there was no significant difference between the suppression in migratory activity of U87 and CNS-1 cells brought on by the inhibitor and that caused by applied flow through the suspensions (*p*>0.05). The baseline migration rates for all cell lines without exposure to shear stress were similar (*p*>0.05; Supplementary Fig. S4). On the other hand, after exposure to shear stress, the U87 cell line migration rates were the lowest among the cell lines, and the CNS-1 cell line migration rates were lower than those of the U251 cell line but remained more invasive than the U87 cell line, while the U251 cell line remained invasive (# *p*<0.015) (Fig. 3*D*).

### Confirmation of TGF-α mediated chemotaxis

**Figure 4:**
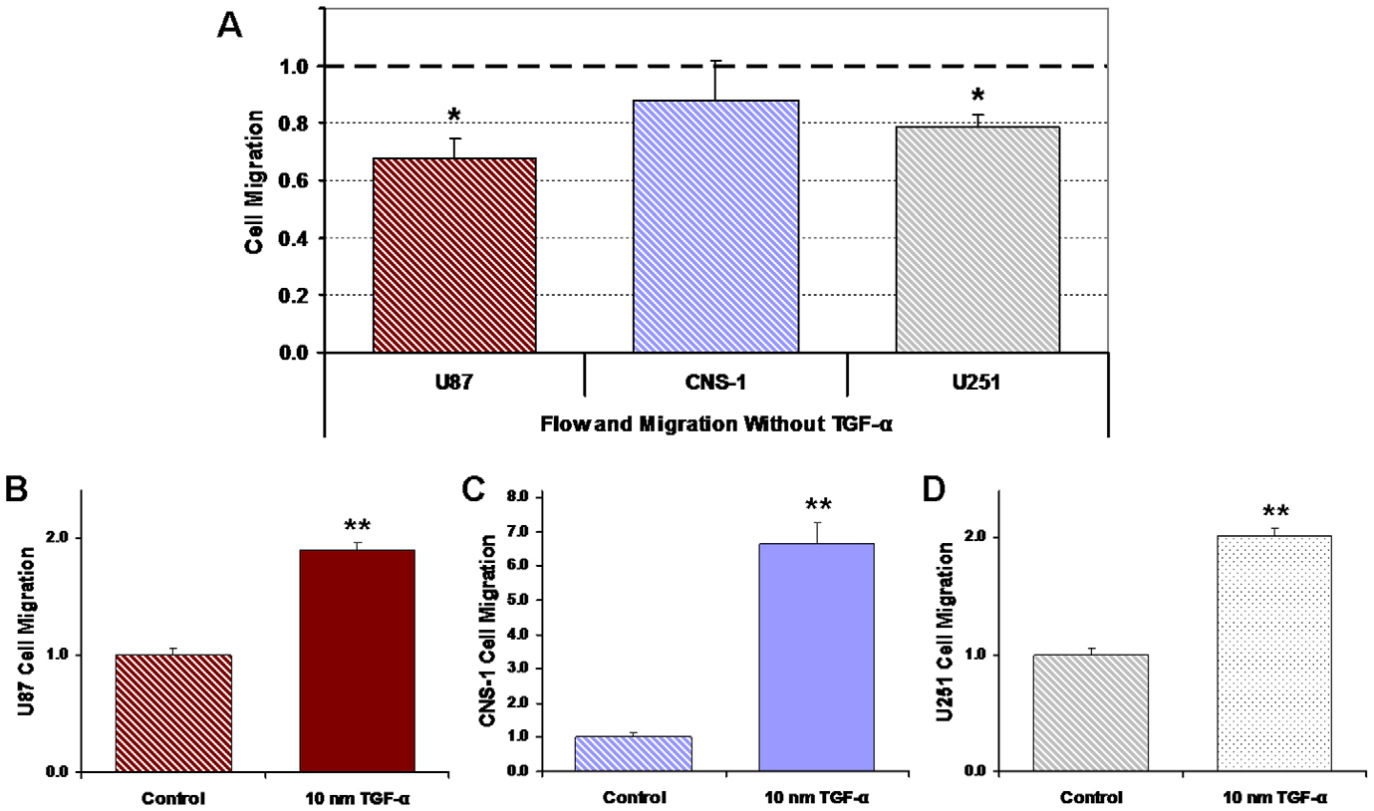
Cell invasion without chemoattractant yielded diminished response of U87, CNS-1, and U251 cells after exposure to flow. All results were normalized to their respective controls (1.0). (**A**) Contrary to expectations of enhanced migration by flow-induced chemokine gradients, suppression of migration persisted in the U87 cell line and small but significant suppression was also observed in the U251 cells. Cells were exposed to a pressure drop of 7 cm H_2_O, 0.55 dynes/cm^2^ shear stress, and invasion assays were conducted without a chemoattractant. (**B–D**) TGF-α effectively directionalized migration of the glioma cell lines; suggests that it was necessary for the quantification of motility and invasive potentials of all the cell lines. Data presented as mean±SEM. Note: * *p*<0.05; ** *p*<0.005.

Cell migration rates without the aid of TGF-α yielded diminished responses for the U87, CNS-1, and U251 cell lines after exposure to flow (Fig. 4*A*). It has been previously shown that flow-induced chemokine gradients can lead to increased cell migration [28]. In this study, however, there was no evidence of enhanced invasion by flow-induced chemokine gradients for any of the cell lines when compared to normalized controls. Though diminished, a 32% suppression of motility was still evident in the U87 cells exposed to 0.55 dynes/cm^2^ shear stress without migration to TGF-α, and though unexpected, there was a 21% suppression in migration of the U251 cells (*p*<0.05). There was no change in the CNS-1 migration rates. Furthermore, TGF-α effectively enhanced the U87, CNS-1, and U251 migratory activity by 89%, 566%, and 101% respectively when compared to normalized controls (*p*<0.005) (Fig. 4*B*–*D*).

### Cell viability and apoptosis in 3D suspensions

**Figure 5:**
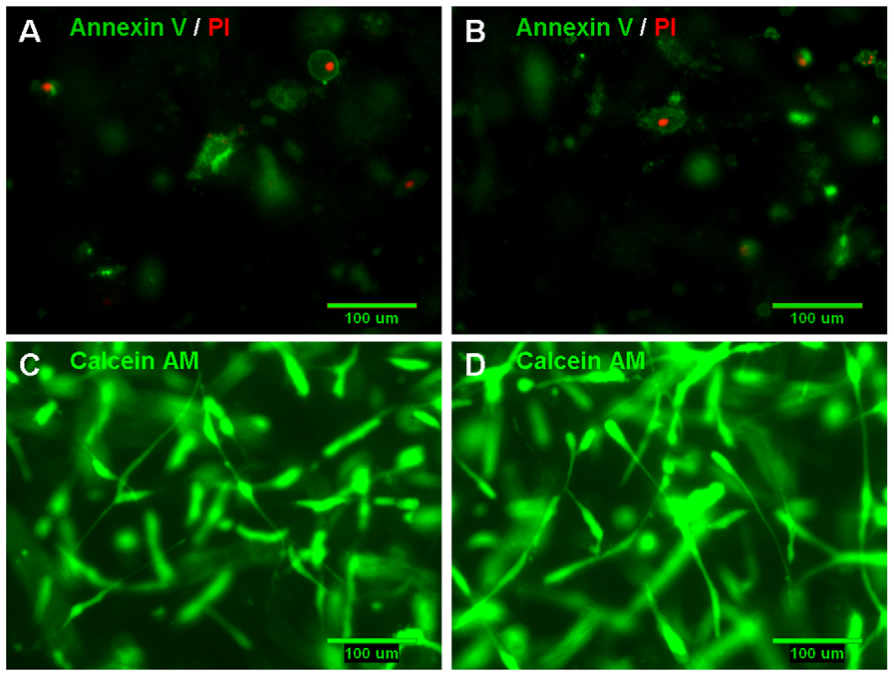
Exposure to shearing forces did not induce cell apoptosis or necrosis. At the end of the migration period, both non-sheared cells (in control gels; **A**, **C**) and cells in gels exposed to 0.55 dynes/cm^2^ shear stress (**B**, **D**) were stained either by the Vybrant Apoptosis Assay Kit no. 2 or by Calcein AM. (**A**, **B**) There was no evidence of apoptosis being induced in the U87 cells as a result of exposure to the higher levels of shearing forces in this experiment; apoptotic cells were stained with Alexa Fluor 488 annexin V (green) and necrotic cells were stained with propidium iodide (red). (**C**, **D**) Calcein AM (green) staining indicates that a majority of the cells remained viable and cell morphology was normal in both gels containing non-sheared U87 cells and cells exposed to 0.55 dynes/cm^2^ shear stress for four hours.

Exposure to four hours of 0.55 dynes/cm^2^ shear stress did not appear to induce cell apoptosis or necrosis in the U87 cell line (Fig. 5*A*–*B*). Calcein AM staining indicated that most of the cells remained viable, morphologically normal, and well spread within both control and flow (sheared) suspensions (Fig. 5*C*–*D*). Similar results were observed with the CNS-1 cell line (Supplementary Fig. S5). The cells that were apoptotic or necrotic deviated from their normal spindle-like appearance to a more contractile morphology. It should be noted that a contractile morphology itself does not imply suppressed migration [38]. A majority of the apoptotic and necrotic cells were observed towards the top of the gel (gel/air interface).

### Downregulation of active and total MMPs

**Figure 6:**
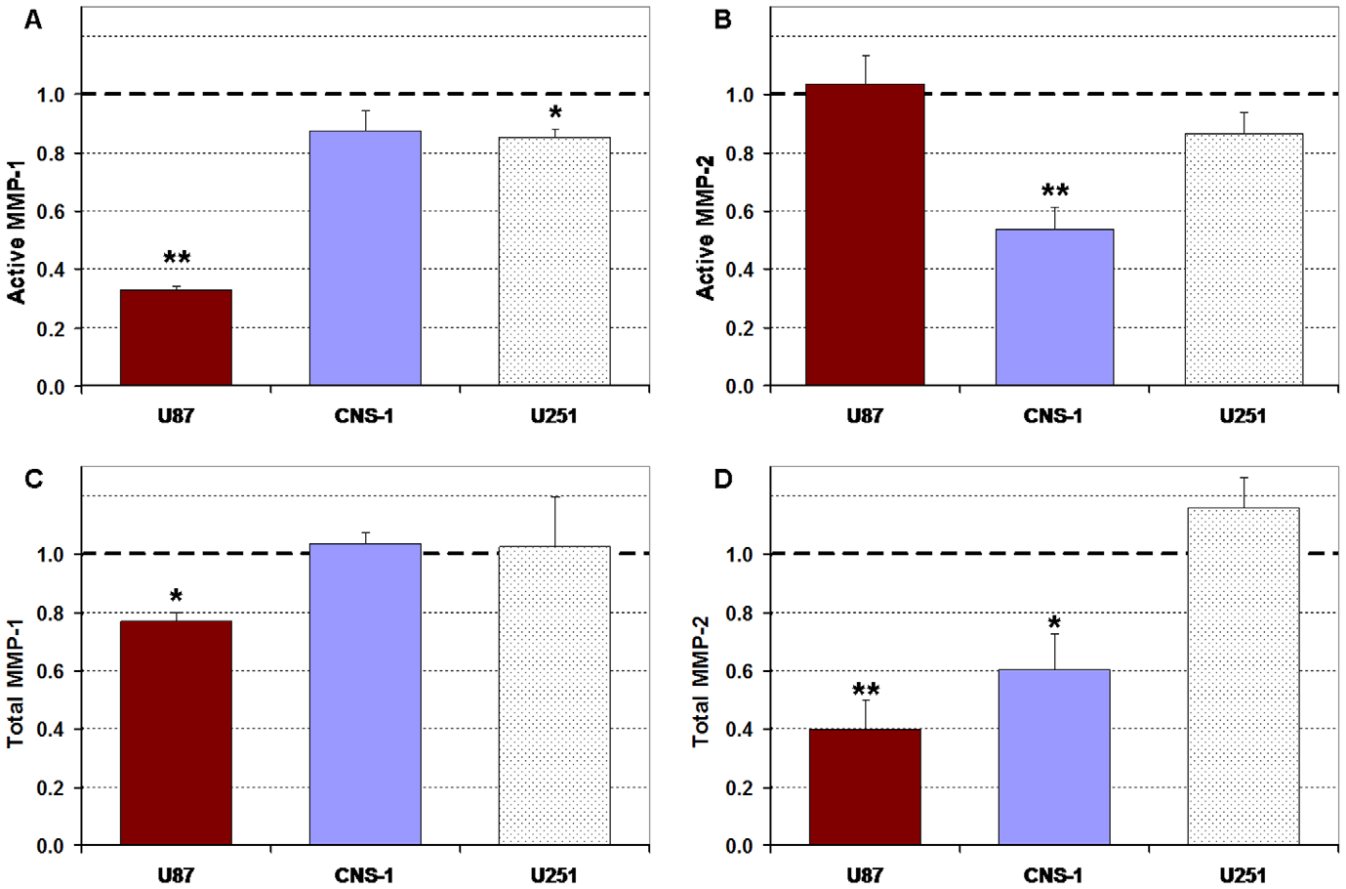
Active and total MMP-1 and MMP-2 levels in conditioned media collected from the wells of inserts containing glioma cells exposed to four hours of 0.55 dyne/cm^2^ sheared and control (non-sheared) cells. All results were normalized to non-sheared controls (1.0). (**A**) Active MMP-1 levels were highly downregulated by shear stress for the U87 cells and slightly reduced for U251 cells (*p*<0.05). (**B**) Shear stress downregulated active MMP-2 levels for the CNS-1 cells (*p*<0.015). (**C**) Total MMP-1 expression was downregulated for the U87 cell line (*p*<0.05). (**D**) Shear stress downregulated total MMP-2 expression for U87 and CNS-1 glioma cell lines (*p*<0.05). Shear affected both collagenase and gelatinase expression for the U87 cells, only gelatinase expression for the CNS-1 cells, and had little effect on the U251 cell line. Data presented as mean±SEM. Note: * *p*<0.05; ** *p*<0.005.

The suppression of U87 migratory activity correlated with downregulation of active MMP-1 production (Fig. 6*A*). Sheared U87 cells (0.55 dynes/cm^2^) exhibited a 68% decrease in active MMP-1 levels when compared to non-sheared cells (*p*<0.005). No significant changes in active MMP-1 levels were observed for the CNS-1 cell line and no significant changes were observed in active MMP-2 levels for the U87 shear cases; however, CNS-1 cells exhibited a 45% decrease in MMP-2 activity (*p*<0.015) (Fig. 6*B*). The suppression of CNS-1 migratory activity correlated with downregulation of active MMP-2 synthesis. U251 cells exhibited a 15% decrease in MMP-1 activity (*p*<0.05) and no significant change in active MMP-2 levels. Sheared U87 cells exhibited a 23% decrease in total MMP-1 and a 60% decrease in total MMP-2; sheared CNS-1 cells exhibited a 40% decrease in total MMP-2 levels and no change in total MMP-1; total MMP-1 and MMP-2 levels for U251 cells were not affected by shear (Fig. 6*C*–*D*).

### Verification of MMP-1 and MMP-2 dependent invasion

**Figure 7:**
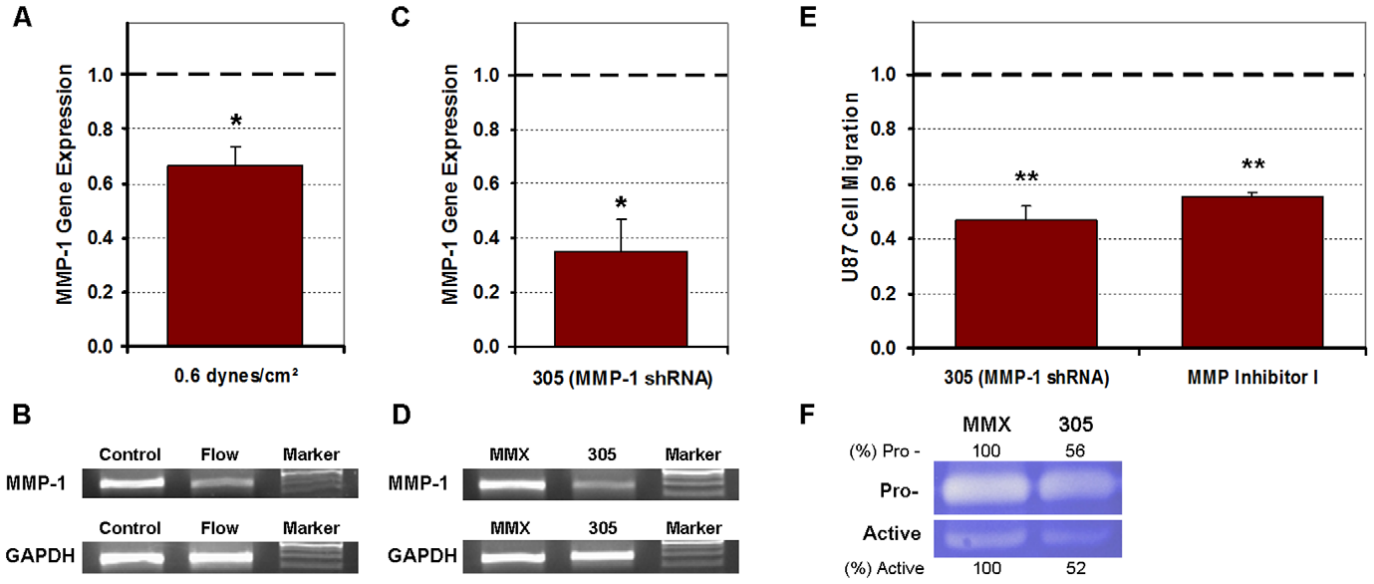
Shear-induced suppression of U87 cell invasive potential was dependent on MMP-1 as determined by PCR, shRNA gene knockdown, and MMP Inhibitor I. All numerical data were normalized to their respective controls (1.0). (**A**) RT-qPCR data indicated that MMP-1 gene expression was downregulated in response to U87 cells being exposed to 0.55 dynes/cm^2^ shear stress. (**B**) Gel electrophoresis of representative samples confirmed that the MMP-1 gene was suppressed in U87 cell suspensions in response to flow (sheared cases) when compared to cells in control (non-sheared) gels. (**C, D**) RT-qPCR and gel electrophoresis of representative samples confirmed gene silencing of MMP-1 in U87 cells transfected with the 305 MMP-1 shRNA compared to cells transfected with the MMX control vector. (**E**) Migration of U87 cells was suppressed when either MMP-1 gene expression was knocked down or when post-translational MMP-1 was targeted by MMP Inhibitor I. (**F**) Collagen zymography of conditioned media collected from the wells of inserts containing U87 cells transfected with 305 shRNA confirmed that both pro- and active MMP-1 were down regulated compared to cells transfected with the MMX control vector. Quantifications for MMP-1 expression are presented as percentage of their respective controls. Data presented as mean±SEM. Note: * *p*<0.05; ** *p*<0.005.

**Figure 8:**
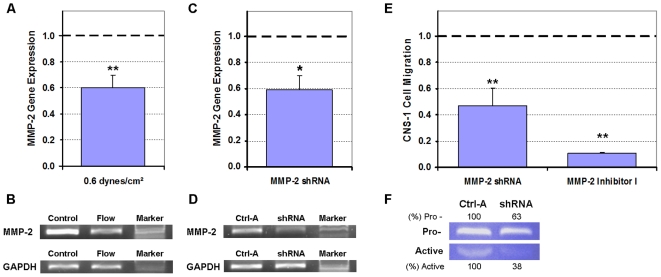
Shear-induced suppression of CNS-1 cell invasive potential was dependent on MMP-2 as determined by PCR, shRNA gene knockdown, and MMP-2 Inhibitor I. All numerical data were normalized to their respective controls (1.0). (**A**) RT-qPCR data indicated that MMP-2 gene expression was downregulated in response to CNS-1 cells being exposed to 0.55 dynes/cm^2^ shear stress. (**B**) Gel electrophoresis of representative samples confirmed that the MMP-2 gene was suppressed in CNS-1 cell suspensions in response to flow (sheared cases) when compared to cells in control (non-sheared) gels. (**C, D**) RT-qPCR and gel electrophoresis of representative samples confirmed gene silencing of MMP-2 in CNS-1 cells transfected with the MMP-2 shRNA compared to cells transfected with the control (Ctrl-A) vector. (**E**) Migration of CNS-1 cells was suppressed when either MMP-2 gene expression was knocked down or when activated MMP-2 was targeted by MMP-2 Inhibitor I. (**F**) Gelatin zymography of conditioned media collected from the wells of inserts containing CNS-1 cells transfected with the MMP-2 shRNA confirmed that both pro- and active MMP-2 were down regulated compared to cells transfected with the control vector. Quantifications for MMP-2 expression are presented as percentage of their respective controls. Data presented as mean±SEM. Note: * *p*<0.05; ** *p*<0.005.

MMP-1 gene expression was downregulated by 33% (*p*<0.05) in U87 cells exposed to 0.55 dynes/cm^2^ shear stress (Fig. 7*A–B*); correlating with the downregulation of total MMP-1 levels. The 305 MMP-1 shRNA, which silenced MMP-1 expression by 65% (*p*<0.05) (Fig. 7*C–D*), diminished the U87 migration by 53% (*p*<0.005) when compared to cells transfected by the MMX control vector (Fig. 7*E*). The MMP Inhibitor I was also able to diminish U87 migratory activity by 45% when compared to normalized controls (*p*<0.005), but was not as potent as the 305 MMP-1 shRNA. Downregulation of MMP-1 expression by shRNA gene silencing was validated by collagen zymography (Fig. 7*F*). In the CNS-1 cells, MMP-2 gene expression was downregulated by 40% (*p*<0.005) after exposure to 0.55 dynes/cm^2^ shear stress (Fig. 8*A–B*); correlating with the downregulation of total MMP-2 levels. The MMP-2 shRNA silenced CNS-1 MMP-2 expression by 41% (*p*<0.05) (Fig. 8*C–D*), and diminished the CNS-1 migration by 53% (*p*<0.005) when compared to cells transfected by the control vector (Fig. 8*E*). The MMP-2 Inhibitor I was more potent than the shRNA and was able to inhibit CNS-1 migratory activity by 89% when compared to normalized controls (*p*<0.005). Downregulation of MMP-2 expression by shRNA gene silencing was validated by gelatin zymography (Fig. 8*F*).

## Discussion

The Darcy Flow experiments necessitated pre-compaction of gels to eliminate cell distribution and gel density differences between the control and sheared cases. The suspensions that were exposed to flow were compacted and this altered the cell distribution such that the flow cases had more cells near the filter than non-compacted controls (Fig. 2*A*). Compacted gels also represent denser ECM that would provide greater resistance to cell migration [39]. A similar phenomenon of compaction by normal stress is observed during the progression of solid tumors when the tumor periphery is exposed to solid stress [40], [41]. The mechanics of confined compaction that were present in this system have been examined by Girton et al [42]. To have valid experimental controls it was necessary for the cell distribution and collagen densities in both the flow cases and control cases to be similar. Based on preliminary experiments it was established that a short application (10 minutes) of flow to compact the control cases to the same level as the flow cases would be ideal. Such cases were designated as compacted controls and were utilized as the experimental controls throughout the study. The cell distribution and gel density of the pre-compacted controls did not differ from those of the gels exposed to flow and ensured the same baseline cell distribution before the migration period (Fig. 2*B*). The methodology of compacting gels is unique to this study; other studies have not characterized cell distribution and compaction of cell suspensions before conducting invasion assays.

The Darcy permeability of the gels was an order of magnitude higher than reported *in vivo*, primarily due to the density of the ECM utilized in this study being lower than that of native tumors. However, the flow velocities, although of the same order of magnitude as *in vivo*, were higher, resulting in shear stress levels applied to the cells within the physiologic range of brain tumors (Table 1). The heterogeneous response to shear stress was pronounced in the flow-modulated motility of the glioma cell lines. Both U87 and CNS-1 cells exhibited time dependent (Supplementary Fig. S3) and shear stress level dependent (Fig. 3*A–B*) suppression in migration stimulated by shear stress. After exposure to a physiologically high level of interstitial fluid shear stress (0.55 dynes/cm^2^), the migratory activity of the U87 cells was essentially eliminated. CNS-1 cell line motility was suppressed as in the U87 cells but to a lesser extent, while the same shear stress levels appeared to have little effect on the migratory activity of the U251 cell line (Fig. 3*C*).

Glioma cells appear to have metastatic morphology/phenotype yet rarely metastasize outside the cranium for reasons that have been thoroughly discussed [43]–[45]. This study suggests that shear stress may contribute to the diminished metastatic and invasive potentials of gliomas. It should be noted that without exposure to flow all cell lines were invasive without distinction in invasive potentials (Supplementary Fig. S4). However, the flow-induced migration trends observed in this study (Fig. 3*D*) are consistent with the reported dissemination of orthotopically implanted tumors of the U87, CNS-1, and U251 cell lines; Candolfi et al demonstrated that CNS-1 cells were more than twice as invasive as the U87 cell line *in vivo*, and that the U251 cells were the most invasive of the three - more than three times as invasive [29].

There are four ways by which interstitial flow can affect cells: flow can apply shear stress on cell surfaces, apply normal force on cell surfaces, apply a tethering force on the extracellular matrix/cell interactions, and form chemical gradients in the direction of flow. The formation of chemical gradients and the application of normal force have been studied through various *in vitro* models [28], [46]. Little is known about flow mediated tethering, however, cell-matrix interactions could amplify shear-induced mechanosensing and tethering forces may be involved in altering local shearing forces through ECM remodeling [26], [47]. In our model, normal force would be expected to enhance migration in the direction of flow, whereas we have observed suppression of migration in the flow direction. In order to eliminate effects associated with convection of signaling molecules and the potential for flow-mediated migration [16], [28], the flow/shear period and the migration (no flow) period were separated, and a chemoattractant gradient utilizing TGF-α was established.

Decoupling the flow period from the migration period effectively depleted any gradients that may have formed during flow, which would be expected to enhance migration, the opposite of shear effects observed here. When invasion assays were performed without a chemoattractant there was no evidence of enhanced invasion by flow-induced chemokine gradients for any of the cell lines, and on the contrary, suppression of migration persisted with the U87 cells and even the U251 cell line had a small attenuation in migration (Fig. 4*A*). Furthermore, TGF-α efficiently established directional migration of the glioma cell lines (Fig. 4*B–D*) indicating that it was essential for the quantification of invasive potentials.

In a prior study with non-tumor cell lines it was determined that a higher differential pressure drop of 10****cmH_2_O across the cell/collagen suspension could induce apoptosis and cell death [26]. However, in this study, exposure to the interstitial flow forces generated by a differential pressure drop of 7****cmH_2_O did not induce cell apoptosis or necrosis in the U87 cell line (Fig. 5) or in the CNS-1 cell line (Supplementary Fig. S5). Contrary to expectations, a majority of the apoptotic/necrotic cells were observed towards the top of the gel. This could have been caused by the application of normal pressure from direct exposure to flow or by effects from the top of the gels being exposed to air for the duration of the migration period. There was no indication for enhanced apoptosis/necrosis towards the gel/filter interface (bottom of the gel).

MMP activity, suppressed via a broad-spectrum MMP inhibitor, was determined to be a dominant mechanism by which glioma cells migrated through the suspensions (Fig. 3*A–C*). Since the suppression of migration by the MMP inhibitor was not significantly different from the suppression of migration by shear stress, modulations in collagenolytic (MMP-1) and gelatinolytic (MMP-2) activities were investigated. It should be noted that applying 10 minutes of flow, for compaction of gels, flushed all the gels with new media which allowed for the removal of proteases that may have accumulated over the initial incubation period. Though additional MMPs may have been flushed out during the four hour flow period, MMPs synthesized over the 48****hour migration period compensated for any dilution effects; MMP assays did not show dilution of MMP levels with respect to no flow controls (Fig. 6). Attenuation in active MMP-1 expression correlated with the shear-induced suppression of U87 migration (Fig. 6*A*). Fluid shear stress induced downregulation of MMP-1 expression and activity has been reported previously in chondrocytes [25]. On the other hand, flow has also been shown to induce upregulation of MMP-13 in vascular smooth muscle cells, the opposite of shear effects observed here [48]. Since MMP-1 levels correlate well with tumor grade and glioma invasiveness [49], flow-induced attenuation in MMP-1 expression implies a diminished invasive potential for U87 gliomas. Attenuation in MMP-2 activity correlated with shear-induced suppression of CNS-1 migration (Fig. 6*B*). Not all tumor cells were susceptible to flow effects; MMP expression in U251 glioma cells appeared to be minimally affected by flow.

Though total (pro- and active) MMP levels do not directly affect migratory activity of cells, invasion in gliomas can be modulated by regulation of pro-enzyme production and activation [30], [50]. It was noteworthy that total MMP expressions declined for U87 and CNS-1 cell lines with exposure to flow suggesting a mechanotransduction pathway that altered synthesis of MMPs within the cells (Fig. 6*C–D*). MMP-1 gene expression was downregulated in U87 cells exposed to shear stress (Fig. 7*A*) which correlated with the downregulation of total MMP-1 levels. Furthermore, both the silencing of MMP-1 gene expression (which reduced both pro- and active MMP-1) and targeting activated MMP-1 diminished U87 cell migration (Fig. 7). The MMP-1 shRNA and the MMP Inhibitor I, however, were not potent enough to suppress U87 migration rates to the level observed in the invasion assays. Therefore, there may have been additional factors such as changes in adhesion molecules or altered cytoskeleton polymerization which contributed to the elevated suppression in motility of the U87 cell line [38], [45]. On the other hand, the MMP-2 shRNA, the broad spectrum MMP Inhibitor and the MMP-2 Inhibitor I were able to match and even surpass the suppression in motility of the CNS-1 cell line (Fig. 8). In the CNS-1 cells, MMP-2 gene expression was attenuated with exposure to shear stress (Fig. 8*A*) which correlated with the downregulation of total MMP-2 levels. Similar to the U87 cells, both the silencing of MMP-2 gene expression (which reduced both pro- and active MMP-2) and inhibiting extracellular MMP-2 diminished CNS-1 cell migration (Fig. 8).

Taken together, the data suggests that the shear-induced suppression in migratory activity was MMP-1 dependent for the U87 cell line and MMP-2 dependent for the CNS-1 cell line. RT-PCR and gene silencing confirmed that interstitial flow was affecting intrinsic levels of specific MMPs suggesting complex mechanisms. The observed flow-induced suppression in MMP expression and decline in motility for U87 and CNS-1 cell lines imply a decrease in invasive potential for glioma cells susceptible to shearing forces.

The present model does not test how invasive potentials may be affected by other factors that exist *in vivo* such as regions of hypoxia, recruitment of stromal cells, and a phenotypically diverse population of cells that are at disparate levels of differentiation [1], [15], [16], [33], [51]. However, hypoxia generates cells with increased migratory activity and has been shown to augment MMP-1 and MMP-2 activities, the opposite of shear effects observed here [1], [14], [33]. Stromal cell are often recruited by tumor cells via extracellular matrix metalloproteinase inducer to enhance invasion and stimulate the production of MMP-1 and MMP-2, again unlike the suppression of migration and MMPs reported here [19], [52]–[55]. Though communications between tumor and host microenvironments have been shown to affect tumor physiology and phenotype [5], little is known about how the interaction between different cell populations within the tumor microenvironment affects invasive potentials. Furthermore, it remains unclear how antiangiogenic therapy and vasculature normalization, which alter the interstitial fluid flow forces [13], [15], would affect the varied invasive potential and growth of tumors.

There are additional models that could be incorporated with the present model to take further advantage of the 3D flow environment [18], [46], [56], [57]. It should be possible to quantify dynamic motility [4] by tracking cells in the 3D model [56]. Cells also could be implanted in a focused area and their invasion into the gel quantified through microscopy [18], [46], [56]. Furthermore, the incorporation of other ECM components including the use of DQ collagen, which contains quenched fluorescence that is released upon collagen degradation, into the current model may reveal additional phenomena [28], [56], [57].

This study displays the heterogeneous migratory behavior of tumor cells in response to fluid shear stress. We have demonstrated that differential invasive potentials may be in part explained by mechanotransduction of flow forces and that fluid shear stress may lower the motility of glioma cells through modulation of MMP activation and expression. It will be of interest to see if interstitial flow forces augment invasive potential in other types of tumors. The models discussed herein are valuable tools for future studies of fluid shear stress effects on the migratory activity of other invasive/non-invasive and metastatic/non-metastatic tumor cell lines, and may be utilized in the identification of novel therapeutics.

## Supporting Information

Figure S1 **Confocal images of cells suspended in collagen gels and stained with Calcein at the end of the 48 hour migration period.** (**A**) Cells remained uniformly distributed within this horizontal slice 50 µm above the filter containing U87 cells exposed to 0.55 dynes/cm^2^ shear stress for 4 hours. (**B**) Cell remained viable and cell morphology was normal in this slice 25 µm above the filter. (**C**) The underside of the 8 µm pore insert filter displays cells that have migrated towards 10 nM TGF-α.
(TIF)

Figure S2 **Fluorescence intensity of gels containing cells stained with Calcein to quantify cell distribution within the collagen suspension.** Fluorescence of the 600 micron thick collagen suspensions (50 µm slices) for control gels that were compacted or not compacted. The cell distribution in non-compacted gels was distinctive when compared to the compacted control gels (# *p*<0.015). The cumulative effect of compaction by flow through the gels was most apparent closer to the filter. In all cases, cells density increased towards the filter interface (*p*<0.0001). All cases were normalized to their respective average intensities and the data presented as mean±SEM.
(TIF)

Figure S3 **Migration response of U87 and CNS-1 cells after time of exposure to shear stress is varied.** The migration responses for cells exposed to the broad spectrum MMP inhibitor were also included. All results were normalized to non-sheared controls (1.0). (**A**) Exposure to 0.55 dynes/cm^2^ shear stress suppressed the migration of U87; (**B**) and CNS-1 cells in a time dependent manner. The U87 and CNS-1 migratory activity was suppressed by up to 92% and 58% respectively when compared to normalized controls (*p*<0.005). The MMP inhibitor suppressed 72% and 86% of the U87 and CNS-1 migratory activity, respectively (*p*<0.005). The suppression of migratory activity in response to the MMP inhibitor was not significantly different from the suppression of migration after increasing time of exposure to shear stress († *p*>0.05). Data presented as mean±SEM. Note: * *p*<0.05; ** *p*<0.005.
(TIF)

Figure S4 **Baseline migration of U87, CNS-1, and U251 glioma cells without exposure to flow (controls).** The baseline migration rates for all cell lines without exposure to shear stress were similar; without exposure to flow all cell lines were invasive without significant differences in invasive potentials (raw migration rates for the three cell lines are presented). Data presented as mean±SEM. Note: † *p*>0.05.
(TIF)

Figure S5 **Exposure to shearing forces did not induce cell apoptosis or necrosis in the CNS-1 cell line.** At the end of the migration period, both non-sheared cells (in control gels; A, C) and cells in gels exposed to 0.55 dynes/cm^2^ shear stress (B, D) were stained either by the Vybrant Apoptosis Assay Kit no. 2 or by Calcein AM. (A, B) There was no evidence of apoptosis being induced in the CNS-1 cells as a result of exposure to the higher levels of shearing forces in this experiment; apoptotic cells were stained with Alexa Fluor 488 annexin V (green) and necrotic cells were stained with propidium iodide (red). (C, D) Calcein AM (green) staining indicates that a majority of the cells remained viable and cell morphology was normal in both gels containing non-sheared CNS-1 cells and cells exposed to 0.55 dynes/cm^2^ shear stress for four hours.
(TIF)
