# Supplementary figures and images for: Fluid Shear Stress Regulates the Invasive Potential of Glioma Cells via Modulation of Migratory Activity and Matrix Metalloproteinase Expression
Source: PLoS One. 2011 May 26;6(5):e20348. doi: 10.1371/journal.pone.0020348 (PMC3102715; doi:10.1371/journal.pone.0020348)

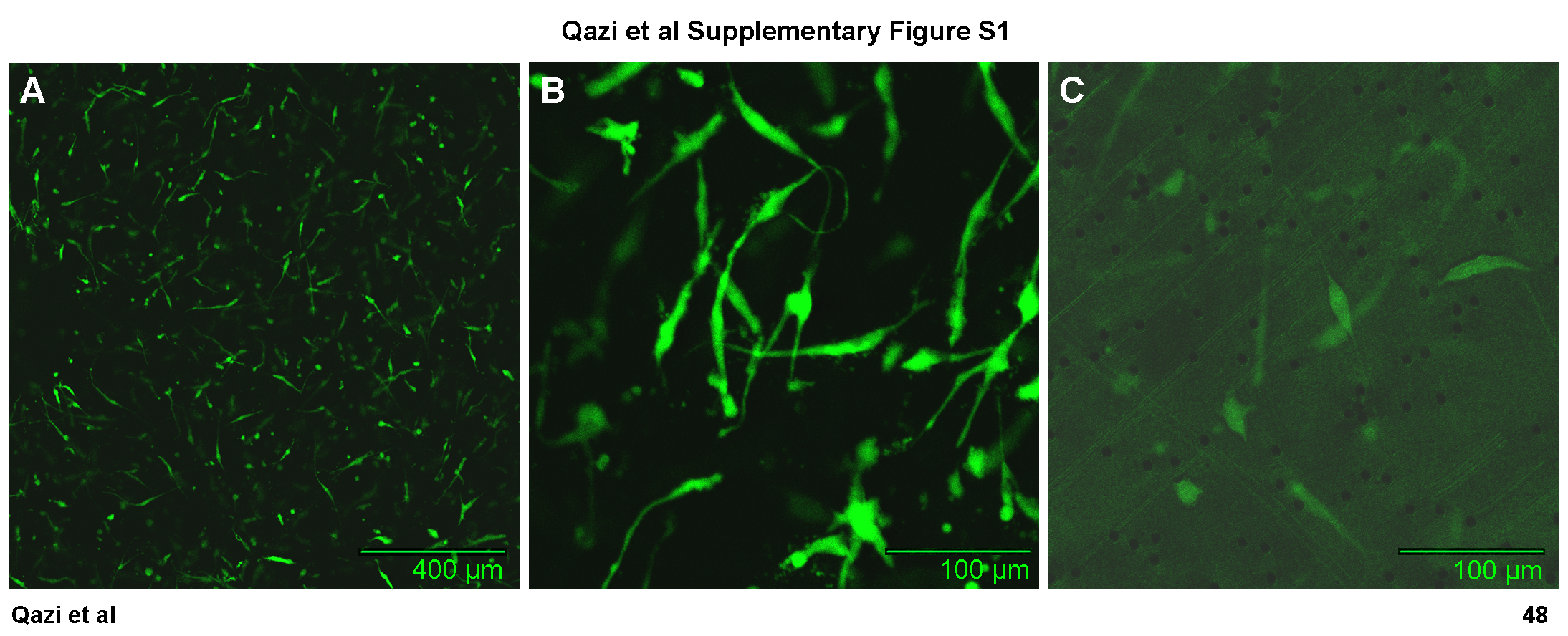

Supplement: Figure S1 — Confocal images of cells suspended in collagen gels and stained with Calcein at the end of the 48 hour migration period. (A) Cells remained uniformly distributed within this horizontal slice 50 µm above the filter containing U87 cells exposed to 0.55 dynes/cm2 shear stress for 4 hours. (B) Cell remained viable and cell morphology was normal in this slice 25 µm above the filter. (C) The underside of the 8 µm pore insert filter displays cells that have migrated towards 10 nM TGF-α. (TIF) [file pone.0020348.s001.tif]

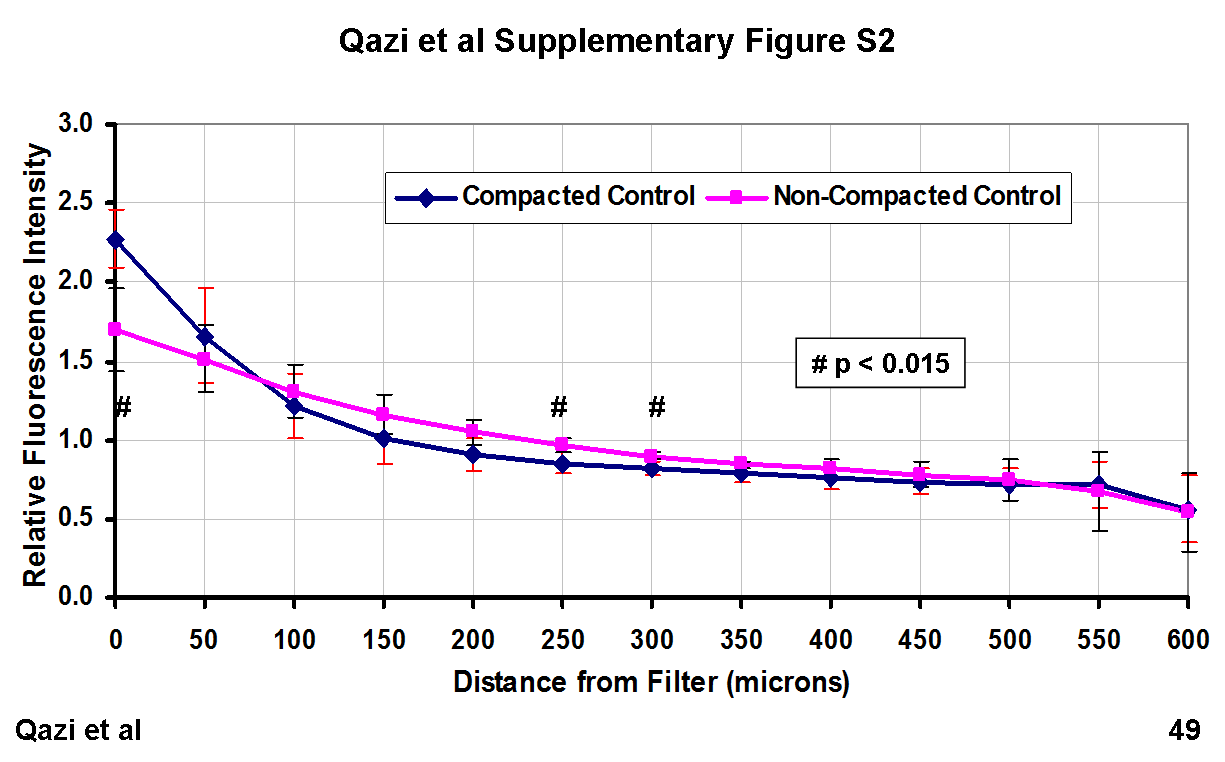

Supplement: Figure S2 — Fluorescence intensity of gels containing cells stained with Calcein to quantify cell distribution within the collagen suspension. Fluorescence of the 600 micron thick collagen suspensions (50 µm slices) for control gels that were compacted or not compacted. The cell distribution in non-compacted gels was distinctive when compared to the compacted control gels (# p<0.015). The cumulative effect of compaction by flow through the gels was most apparent closer to the filter. In all cases, cells density increased towards the filter interface (p<0.0001). All cases were normalized to their respective average intensities and the data presented as mean±SEM. (TIF) [file pone.0020348.s002.tif]

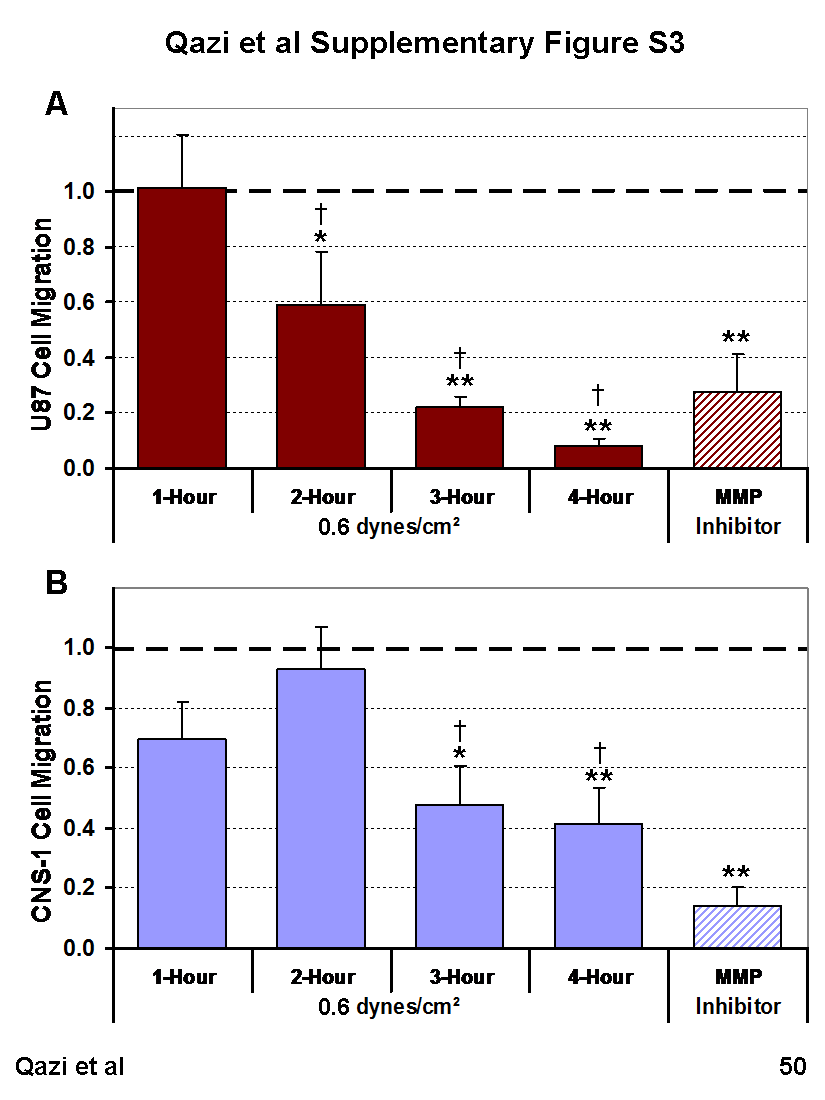

Supplement: Figure S3 — Migration response of U87 and CNS-1 cells after time of exposure to shear stress is varied. The migration responses for cells exposed to the broad spectrum MMP inhibitor were also included. All results were normalized to non-sheared controls (1.0). (A) Exposure to 0.55 dynes/cm2 shear stress suppressed the migration of U87; (B) and CNS-1 cells in a time dependent manner. The U87 and CNS-1 migratory activity was suppressed by up to 92% and 58% respectively when compared to normalized controls (p<0.005). The MMP inhibitor suppressed 72% and 86% of the U87 and CNS-1 migratory activity, respectively (p<0.005). The suppression of migratory activity in response to the MMP inhibitor was not significantly different from the suppression of migration after increasing time of exposure to shear stress († p>0.05). Data presented as mean±SEM. Note: * p<0.05; ** p<0.005. (TIF) [file pone.0020348.s003.tif]

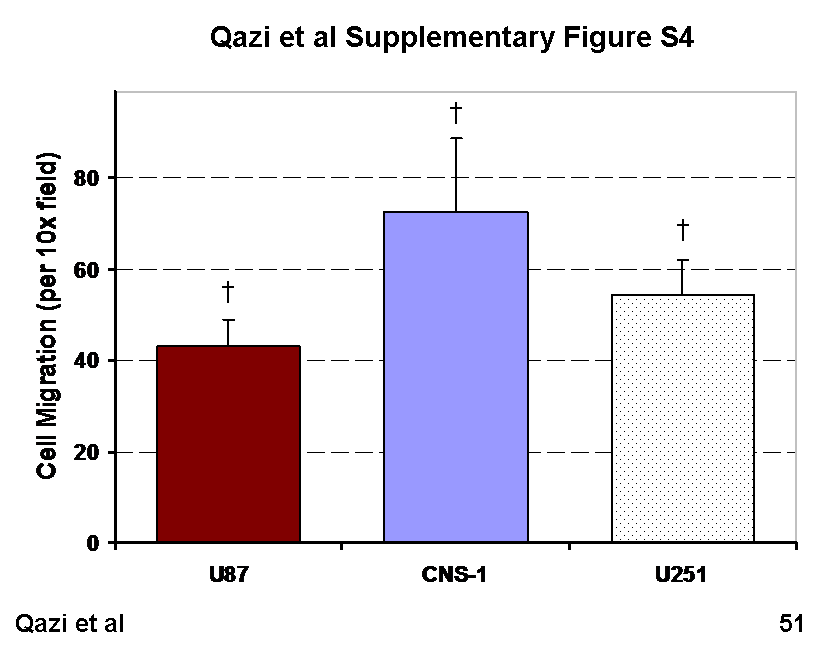

Supplement: Figure S4 — Baseline migration of U87, CNS-1, and U251 glioma cells without exposure to flow (controls). The baseline migration rates for all cell lines without exposure to shear stress were similar; without exposure to flow all cell lines were invasive without significant differences in invasive potentials (raw migration rates for the three cell lines are presented). Data presented as mean±SEM. Note: † p>0.05. (TIF) [file pone.0020348.s004.tif]

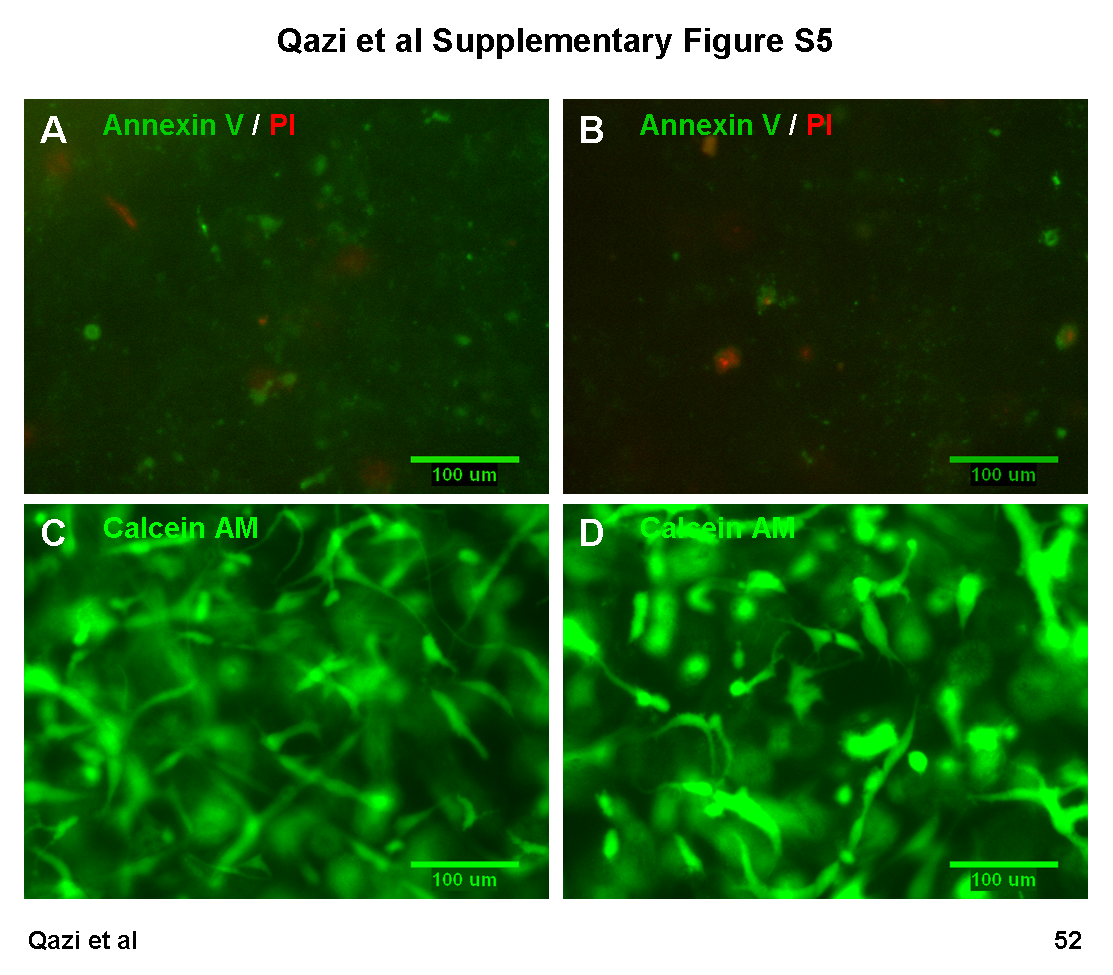

Supplement: Figure S5 — Exposure to shearing forces did not induce cell apoptosis or necrosis in the CNS-1 cell line. At the end of the migration period, both non-sheared cells (in control gels; A, C) and cells in gels exposed to 0.55 dynes/cm2 shear stress (B, D) were stained either by the Vybrant Apoptosis Assay Kit no. 2 or by Calcein AM. (A, B) There was no evidence of apoptosis being induced in the CNS-1 cells as a result of exposure to the higher levels of shearing forces in this experiment; apoptotic cells were stained with Alexa Fluor 488 annexin V (green) and necrotic cells were stained with propidium iodide (red). (C, D) Calcein AM (green) staining indicates that a majority of the cells remained viable and cell morphology was normal in both gels containing non-sheared CNS-1 cells and cells exposed to 0.55 dynes/cm2 shear stress for four hours. (TIF) [file pone.0020348.s005.tif]
